# Improved Denoising of Structural Vibration Data Employing Bilateral Filtering

**DOI:** 10.3390/s20051423

**Published:** 2020-03-05

**Authors:** Ning Liu, Thomas Schumacher

**Affiliations:** 1 School of Civil Engineering, Jilin Jianzhu University, Changchun 130118, China; liuning86@jlju.edu.cn; 2 Department of Civil and Environmental Engineering, Portland State University, Portland, OR 97201, USA

**Keywords:** structural vibrations, denoising, bilateral filtering, structural damage detection, time-frequency analysis, signal-to-noise ratio, time-frequency spectrum energy, magnitude-squared coherence

## Abstract

With the continuous advancement of data acquisition and signal processing, sensors, and wireless communication, copious research work has been done using vibration response signals for structural damage detection. However, in actual projects, vibration signals are often subject to noise interference during acquisition and transmission, thereby reducing the accuracy of damage identification. In order to effectively remove the noise interference, bilateral filtering, a filtering method commonly used in the field of image processing for improving data signal-to-noise ratio was introduced. Based on the Gaussian filter, the method constructs a bilateral filtering kernel function by multiplying the spatial proximity Gaussian kernel function and the numerical similarity Gaussian kernel function and replaces the current data with the data obtained by weighting the neighborhood data, thereby implementing filtering. By processing the simulated data and experimental data, introducing a time-frequency analysis method and a method for calculating the time-frequency spectrum energy, the denoising abilities of median filtering, wavelet denoising and bilateral filtering were compared. The results show that the bilateral filtering method can better preserve the details of the effective signal while suppressing the noise interference and effectively improve the data quality for structural damage detection. The effectiveness and feasibility of the bilateral filtering method applied to the noise suppression of vibration signals is verified.

## 1. Introduction

### 1.1. Motivation

Damage detection is the first step in the non-destructive evaluation (NDE) of structures [1]. Thanks to the advancements in hard- and software, and signal processing, vibration-based structural damage detection can be achieved having only the structural response data to estimate the health of a structure [1]. For practical applications on in-service structures, due to the smallness and/or complexity of structural damage, the relatively small vibration response amplitudes, and the unpredictability of the environmental excitations, the recorded response signals are often non-stationary and contain noise [1]. Thus, the extraction of signal components that contain information regarding structural damage from the collected complex response signal becomes a critical step in the identification of structural damage [2]. Note that in structural damage detection, noise refers to unrelated signal content that does not reflect structural vibration characteristics. This noise can be of significant amplitude, making structural damage detection difficult or impossible. Consequently, discriminating and eliminating unwanted signal content such as noise is the first critical step in structural damage detection. The objective of effective denoising is thus to maximize the vibration-related information of the structure in order to enable an effective data analysis and achieve reliable and rational structural damage detection [3,4]. 

### 1.2. Background

To date, a number of denoising algorithms have been developed and used for the denoising of structural vibration signals, such as moving average filter [5], median filter [6], Kalman filter [7], and more. Unfortunately, these filters are often not effective or can be even detrimental for practical applications. For example, a moving average filter dose not denoise effectively when the signal contains wide-band frequency noise [5]. The median filter has been found to be effective for suppressing large-amplitude pulse interference, but some details of the signal content of interest may be suppressed [6,7]. Wavelet analysis is suitable for analyzing nonlinear signals because the analysis takes place over a portion of a signal, i.e., on a window with adjustable length, in the time and frequency domains [8]. Mathematically, wavelet denoising is a function approximation problem in which the best approximation of the measured signal is sought. Signal denoising is achieved by expansion and translation of a wavelet basis function using a certain measurement criterion, which can be treated as an optimization problem [8,9,10]. Donoho and Johnstone proposed the wavelet threshold denoising technique based on the wavelet transform using both hard as well as soft threshold function methods [11,12,13,14]. While these two threshold denoising methods work well for some signals, they have deficiencies and shortcomings due to the discontinuity of the wavelet coefficients and their associated ability to reconstruct the measured signals.

Unlike the preceding denoising methods, singular value decomposition (SVD) can realize signal noise suppression by dividing a noise-contaminated signal into signal and subspaces [15]. Zhao and Ye researched the principles of Hankel matrix-based SVD and wavelet transform in noise suppression, and a singularity detection experiment was conducted to test their performances [16]. Feng and Dong applied SVD to vibration signals by the construction of the singular space, which was divided into singular and noise singular value subspaces according to the distribution of the singular value subspace. The noise singular value subspace was removed by setting a proper threshold [15]. Empirical mode decomposition (EMD) is a time-frequency analysis method developed by Huang et al. [17] to process complex signals. EMD can deal with nonlinear and non-stationary signals; hence, it has a large number of applications in vibration data processing. Boudraa and Cexus introduced an EMD-based noise reduction method. In their study, the noisy signal was first decomposed adaptively into intrinsic mode functions (IMFs). By filtering or thresholding each IMF, an estimated signal was reconstructed from the processed IMFs [18]. Fan and Zhencai combined EMD and SVD to process noisy vibration signals in their research. EMD was applied to decompose the obtained signals into IMFs, then only IMFs including the characteristic damage frequencies (CDFs) and higher frequency components were selected to do further noise reduction using SVD; other IMFs were classified as noise and abandoned [19].

### 1.3. Significance and Aims

Ideally, denoising preserves signal content of interest while rejecting noise. This is of particular importance in structural health monitoring (SHM) applications where field data often has a low signal-to-noise ratio (SNR). To achieve this goal for image processing, researchers have proposed a number of local adaptive algorithms. Tomasi and Manduchi proposed a non-iterative algorithm for image denoising, referred to as bilateral filtering algorithm [20]. Compared with traditional iterative algorithms, the bilateral filtering algorithm better preserves the image edge information while eliminating image noise and thus achieves more effective denoising. Paris and Kornprobst comprehensively reviewed and summarized the bilateral filtering algorithm and applied it to image denoising, extraction, and texture, as well as tone adjustment [21,22]. Although bilateral filtering algorithms have been widely used in image processing, including medical tomography and nuclear magnetic resonance scanning, their application to structural vibration data is novel. In this article, we evaluated bilateral filtering for denoising of structural vibration data and demonstrated its ability to provide superior denoising and ultimately promise improvement for damage detection tasks. For our evaluation, we used both synthetically generated signals as well as signals collected from a small-scale laboratory experiment.

### 1.4. Article Outline

The theoretical basis of bilateral filtering is introduced in Section 2. Means for calculating the SNR, a time-frequency analysis method called *S* transform, and a method to quantify the time-frequency spectrum-*Frobenius* norm were introduced to evaluate the denoising effects. In Section 3, numerical examples are employed to demonstrate the effectiveness of the proposed technique and denoising effects are compared with other methods. The effectiveness of the proposed bilateral filtering technique is verified on experimental data in Section 4. Finally, conclusions are drawn and recommendations for further work are presented in Section 5.

## 2. Methodologies

Section 2.1 introduces the concept of bilateral filtering and how it is applied to structural vibration time series data. Section 2.2, Section 2.3 and Section 2.4 define methods to characterize the effects this filter has on a time series.

### 2.1. Theory of Bilateral Filtering with Illustrative Example

**Figure 1:**
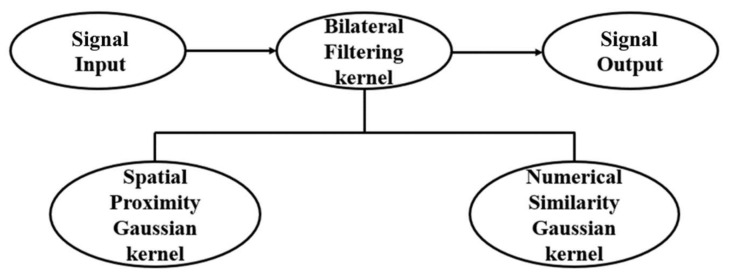
Schematic of a bilateral filtering process.

Bilateral filtering is a nonlinear, non-iterative low-pass filtering technique commonly used for edge-preserving image smoothing [15]. It employs a bilateral filter kernel function by multiplying the spatial proximity Gaussian kernel and the numerical similarity Gaussian kernel functions. Current data points are replaced by those obtained by weighting the neighborhood data points, thereby achieving the purpose of filtering. The process of bilateral filtering is illustrated in Figure 1. 

The two Gaussian weights in the bilateral filtering technique are related to spatial distance and numerical similarity. The expression only considering the spatial weight Gaussian filter is [20]:

(1) $$h(x_{ij})=k_{d}^{-1}(x_{ij})\int_{-\infty }^{\infty }\int_{-\infty }^{\infty }\xi_{st}c((s,t),(i,j))dsdt$$

where

(2) $$k_{d}(x_{ij})=\int_{-\infty }^{\infty }\int_{-\infty }^{\infty }c((s,t),(i,j))dsdt$$

where (s,t) and (i,j) are two pixels, $c\left(\right)$ is the Gaussian weight function based on spatial distance, and $\xi_{st}$ and $x_{ij}$ are the gray levels of (s,t) and (i,j), respectively. $k_{d}(x_{ij})$ is used to normalize the output signal.

Gaussian filtering as defined by Equation (1) is effective as a low-pass filter, but one problem is that only spatial distance information of the data is considered. The filtered signal thus experiences a loss of edge information. Edge in this article mainly refers to the instances in which numerical amplitude values suddenly change. Bilateral filtering solves this problem by adding another weight function to the Gaussian filter. The expression of a Gaussian filter considering numerical weights is

(3) $$h(x_{ij})=k_{r}^{-1}(x_{ij})\int_{-\infty }^{\infty }\int_{-\infty }^{\infty }\xi_{st}s(\xi_{st},x_{ij})dsdt$$

where

(4) $$k_{r}(x_{ij})=\int_{-\infty }^{\infty }\int_{-\infty }^{\infty }s(\xi_{st},x_{ij})dsdt$$

where $s\left(\right)$ is the Gaussian weight function based on numerical similarity and $k_{r}(x_{ij})$ is used to normalize the output signal. By combining the two expressions, a bilateral filtering expression based on spatial distance and numerical similarity is obtained, as follows:

(5) $$h(x_{ij})=k_{}^{-1}(x_{ij})\int_{-\infty }^{\infty }\int_{-\infty }^{\infty }\xi_{st}c((s,t),(i,j))s(\xi_{st},x_{ij})dsdt$$

where

(6) $$k(x_{ij})=\int_{-\infty }^{\infty }\int_{-\infty }^{\infty }c((s,t),(i,j))s(\xi_{st},x_{ij})dsdt$$

Equation (6) combines two Gaussian weight functions, $k_{d}(x_{ij})$ and $k_{r}(x_{ij})$ (Equations (2) and (4)) where c((s,t),(i,j)) and $s(\xi_{st},x_{ij})$ are measures for the spatial and numerical similarity between the center pixel $x_{ij}$ and its neighbor $\xi_{st}$, respectively. Usually, these two measures can be defined as two Gaussian Kernel functions:

(7) $$c((s,t),(i,j))=e^{-\frac{1}{2}{\left(\frac{\left\|\left(s,t)-(i,j\right)\right\|}{\sigma_{d}}\right)}^{2}}$$

(8) $$s(\xi_{st},x_{ij})=e^{-\frac{1}{2}{\left(\frac{\left\|\xi_{st}-x_{ij}\right\|}{\sigma_{r}}\right)}^{2}}$$

The bilateral filter integrates spatial and numerical similarity filters into one. It defines a space neighborhood using spatial filter variance, $\sigma_{d}$. The numerical similarity filter is used for selecting the points with the similar gray levels in the defined spatial neighborhood.

Equations (1) to (8) are infinite integrals in space; however, they need to be discretized before they can be employed in a numerical algorithm. This article considers the case where a signal is one-dimensional, e.g., the acceleration response time-history from a structural vibration test. To illustrate the capabilities of bilateral filtering, an example is presented next. Consider a signal, X representing the noise-free version of a response signal. Zero-mean random Gaussian noise, N is added to signal,X, producing a more realistic real-world response signal, Y:

(9) Y=X+N

The bilateral filtering technique restores the original signal, X by weighted averaging the amplitudes in the response signal, Y:

(10) $$X[\xi ]=\frac{\sum_{x=-R}^{R}W[\xi ,x]Y[\xi -x]}{\sum_{x=-R}^{R}W[\xi ,x]}$$

where R is the smoothing distance (or radius). Equation (10) represents a normalized weighted average of 2R+1 sized neighborhood points centered at the $\xi^{th}$ sampling point in Y to obtain the filtered signal amplitude at the $x^{th}$ sampling point. The weight coefficients function, W[ξ,x] is the product of the two factors based on the spatial distances weight function, $W_{d}[\xi ,x]$ and the numerical similarity weights function, $W_{r}[\xi ,x]$, which are defined as follows:

(11) $$W_{d}[\xi ,x]=e^{-d^{2}([\xi ],[\xi -x])/2\sigma_{d}^{2}}=e^{-x^{2}/2\sigma_{d}^{2}}$$

(12) $$W_{r}[\xi ,x]=e^{-d^{2}(Y[\xi ],Y[\xi -x])/2\sigma_{r}^{2}}=e^{-{\left(Y[\xi ]-Y[\xi -x]\right)}^{2}/2\sigma_{r}^{2}}$$

where d represents the distance measure operator.

**Figure 2:**
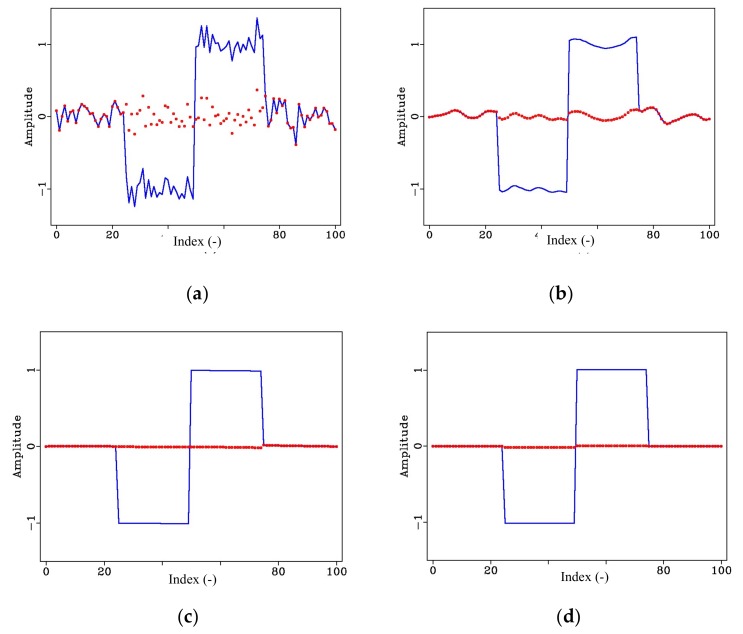
Effect of bilateral filter on synthetic signal with three different sets of parameters: (**a**) Synthetic square wave signal with added random noise; (**b**) filtered signal using R= 3, $\sigma_{d}$ = 5, and $\sigma_{r}$ = 0.05; (**c**) filtered signal using R = 5, $\sigma_{d}$ = 10, and $\sigma_{r}$ = 0.1, and (**d**) filtered signal using R = 15, $\sigma_{d}$ = 10, and $\sigma_{r}$ = 0.1. The dotted red line represents the residual noise.

As can be observed from the equations above, the bilateral filter is controlled by three parameters: the smoothing radius, R, where larger values provide stronger smoothing, but at the same time causes data distortion, the spatial distance standard deviation $\sigma_{d}$, and the numerical similarity standard deviation, $\sigma_{r}$, which determine the degree of attenuation of the two weighting functions, $W_{d}[\xi ,x]$ and $W_{r}[\xi ,x]$, respectively. For large values of these parameters, bilateral filtering converges with mean filtering. On the other hand, if the values for the three parameters are too small, the smoothing effect is weakened. Figure 2 illustrates the effect of bilateral filtering using three different sets of parameters on a synthetic noisy square wave signal, the dotted red lines in each figure represent the residual noise.

### 2.2. Reference Filtering Methods

In this article, we used median filtering and wavelet filtering as two classic noise suppression methods for comparison with our proposed bilateral filter. Subsequently, we introduce these two methods briefly.

Median filtering is a non-linear signal processing method particularly used for signal and image smoothing [6]. It is performed by letting a window with a select number of points slide over a signal and replace the value at the window center with the median of the original values within the window. This process produces an output sequence that usually is smoother than the original one. Median filtering is a simple but effective method for smoothing signals with random spikes with minimal interference of the actual features.

Wavelet denoising is another effective non-linear technique that operates on one wavelet coefficient at a time [8]. In its most basic form, each coefficient is compared against a threshold. If the coefficient is smaller than threshold, it is set to zero; otherwise, it is kept or modified. Replacing all small noisy coefficients by zero and the performing an inverse wavelet transform on the result may lead to a reconstruction with the essential signal characteristics and less noise. Wavelet denoising involves three steps: (1) a linear discrete wavelet transform, (2) a nonlinear thresholding step, and (3) a linear inverse wavelet transform. 

### 2.3. Signal-to-Noise Ratio (SNR)

Several sources of noise exist in recorded real-world structural vibration signals that need to be considered, including environmental and ambient noise, noise from the measurement device itself, noise from the test setup, etc. For the synthetic signals used in this article, high-frequency white noise following a Gaussian distribution was used. To estimate the signal-to-noise ratio (SNR), the superposition method is used [23,24]. In the SNR analysis, window D, the data volume is recorded as:

(13) $$D={\left[d_{i}\right]}_{M}$$

where M is the number of sampling points in the time window analyzed, i=1,2,⋯,M. If the noise is randomly distributed at zero mean and is independent of the signal along the measurement direction, then

(14) $$d_{i}=s_{i}+n_{i}$$

where $s_{i}$ and $n_{i}$ are signal and noise amplitudes at index *i*, respectively. Furthermore,

(15) $$\sum_{i=1}^{M}n_{i}=0$$

The energy of the signal containing noise is

(16) $$E_{S}=\sum_{i=1}^{M}s_{i}{}^{2}=\frac{1}{M}{\left(\sum_{i=1}^{M}d_{i}\right)}^{2}$$

The energy of the noise is

(17) $$E_{N}=\sum_{i=1}^{M}d_{i}{}^{2}-E_{S}$$

The resulting expression for the SNR is

(18) $$SNR=\frac{E_{S}}{E_{N}}=10\log_{10}\left(\frac{\sum_{i=1}^{M}d_{i}{}^{2}}{M\sum_{i=1}^{M}d_{i}{}^{2}-\sum_{i=1}^{M}d_{i}{}^{2}}\right)$$

### 2.4. Theory of the S Transform

While the Fourier transform (FT) contains information about the spectral components of a time series, the temporal distribution of them is lost. Hence, for the analysis of real world, non-stationary signals, the FT may have limited use. In order to study the local properties of the signal in the time and frequency domains simultaneously, Gabor proposed a windowed FT, also known as short time Fourier transform (STFT) [25]. The STFT is now widely used, but due to the limitation of the fixed window length, the time and frequency resolutions are restricted mutually and do not have adaptability. The Wavelet transform (WT) adapts the STFT localization idea and overcomes some shortcoming by offering flexible window lengths [26]. However, the disadvantage of the WT is that there is no direct relationship between wavelet series and frequency.

In 1996, geophysicist Stockwell proposed the *S* transform (ST) based on previous time-frequency analysis studies [27]. The ST combines the advantages of the STFT and WT. The reciprocal of the frequency in the ST determines the size of the Gaussian window scale, and it possesses the advantages of the multi-resolution property of the WT. Furthermore, there is a phase factor in the ST that preserves each of the absolute phase characteristics of the frequency, which is not available in the WT. The one-dimensional continuous ST is defined as follows:

(19) $$S(\tau ,f)=\int_{-\infty }^{\infty }f(t)\frac{\left|f\right|}{\sqrt{2\pi }}e^{\left[\frac{-f^{2}{\left(\tau -t\right)}^{2}}{2}\right]}e^{-2\pi ft}dt$$

where *S* denotes the ST of the time series, f(t), f and t represent frequency and time, respectively, and τ controls the position of the Gaussian window on the time axis, which is equivalent to the shift factor in the WT.

### 2.5. Energy of the Spectrum

The ST produces the time-frequency spectrum of a time series such as acceleration measured from structural vibrations. In order to quantify the change in energy of the time-frequency spectrum, we introduce the concept of the *Frobenius* norm (*F*-norm) [28]. The *F*-norm can be defined in the following different ways:

(20) $${\left\|A\right\|}_{F}=E=\sqrt{\sum_{i=1}^{m}\sum_{j=1}^{n}{\left|a_{ij}\right|}^{2}}=\sqrt{trace(A^{*}A)}=\sqrt{\sum_{i=1}^{\min\{m,n\}}\sigma_{i}^{2}}$$

where $A^{*}$ represents the conjugate transpose of A, $\sigma_{i}$ is the singular value of A. The *F*-norm is similar to the Euclidean norm representing the inner product from the space of all matrices. For the time-frequency spectrum S(t,f) obtained based on the ST, the energy, E of the time-frequency spectrum can be expressed as follows:

(21) $$E=\sqrt{\sum_{i=1}^{m}\sum_{j=1}^{n}S^{2}(t_{i},f_{j})}$$

where m and n are the time-frequency spectrum pixel points in the time and frequency directions respectively, can be defined by m=T⋅Pt, n=F⋅Pf. *T* and *F* are the ranges of time and frequency directions in the time-frequency spectrum, Pt and Pf are time and frequency resolution, respectively.

## 3. Evaluation Using Synthetic Signals

In order to illustrate the capabilities of bilateral filtering for processing one-dimensional time series, two examples using synthetic signals are discussed in this section: a dual-frequency chirp signal and a structural damped free vibration signal. Gaussian white noise with a variance of 10% was added to both signals. Median filtering, wavelet denoising, and bilateral filtering are applied to the signals and their ability to suppress noise while retaining signal features of interest is compared.

### 3.1. Dual-Frequency Chirp Signal

**Figure 3:**
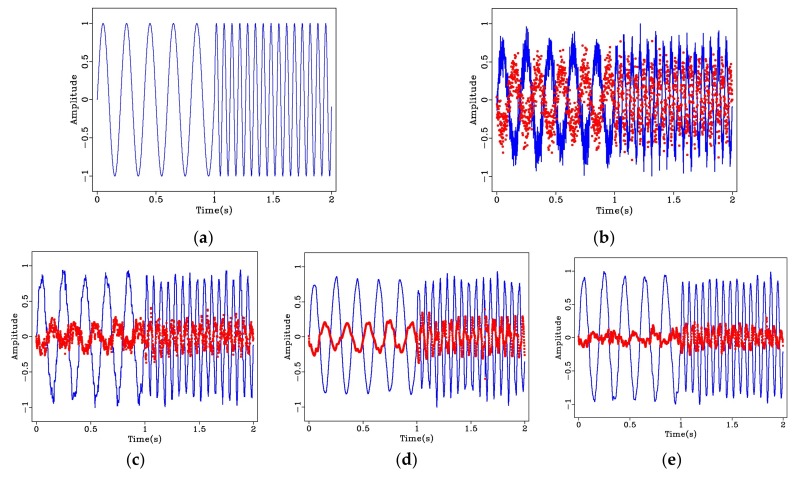
Synthetic dual-frequency chirp signal and denoising effects: (**a**) Original signal; (**b**) original noisy signal; (**c**) signal using median filtering; (**d**) signal using wavelet denoising, and (**e**) signal using bilateral filtering. The dotted red line represents the residual noise.

Figure 3a shows a chirp signal with a length of 2 s and a sampling frequency of 1000 Hz, having two frequency components of 5 and 15 Hz. The signal after adding random noise as shown in Figure 3b. Although the period of the original signal is retained, the noise greatly changes the amplitude of the original signal. The effect of the median filter for noise suppression is shown in Figure 3c. It can be observed that, while some impulse noise interferences are removed, some signal loss is introduced, resulting in a relatively large difference between the filtered and the original noise-free signal. The signal after employing wavelet denoising is shown in Figure 3d. While this technique shows some improvement, there is still distortion in the high frequency portion of the signal. The result from bilateral filtering is displayed in Figure 3e. It can be observed that the denoised signal is closer to the original signal both in terms of the smoothness and variation of amplitudes. The parameters used are: R = 15, $\sigma_{d}$ = 10, and $\sigma_{r}$ = 0.1. The dotted red line represents the residual noise. It can be seen from the Figure 3b to Figure 3e, the Bilateral Filtering method obtained the best denoising result.

By calculating the SNR, the effects of the used denoising techniques can be compared quantitatively. As shown in Table 1, both median filtering as well as wavelet denoising improve the SNR of the original noisy signal. Bilateral filtering, however, has the highest SNR compared to the other methods for the signal studied.

**Figure 4:**
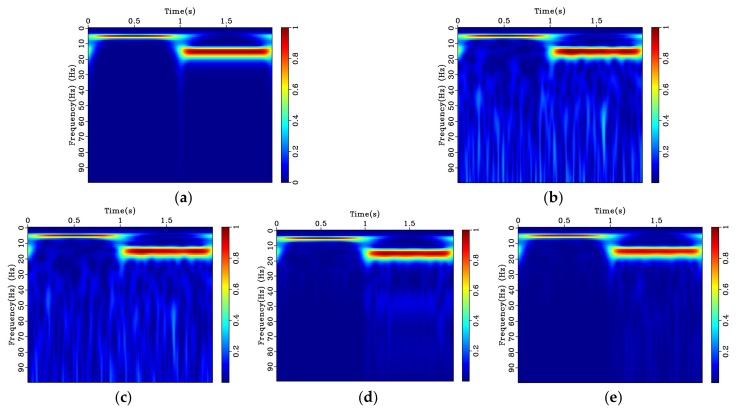
Time-frequency spectrum based on S transform for simulated chirp signal with random noise and denoising results: (**a**) Original signal; (**b**) original noisy signal; (**c**) signal using median filtering; (**d**) signal using wavelet denoising, and (**e**) signal using bilateral filtering.

To illustrate the applicability and superiority of the proposed bilateral filtering technique, time-frequency analysis based on the *S* transform is performed. Figure 4a accurately characterizes the two frequencies of the chirp signal. It can be observed from the time-frequency spectrum after adding the noise (Figure 4b), that the amplitude value of the noise is spread over the entire frequency band, with noise amplitudes gradually increasing from 30 to 90 Hz. The median filtering result is shown in Figure 4c. It can be observed that the amplitude of the noise across the whole frequency band is only minimally reduced. The wavelet denoising result presented in Figure 4d shows significant improvement over the low frequency portion of the signal, but there is still noise interference in the high frequency portion. Bilateral filtering displayed in Figure 4e while still not perfect, shows further improvement as the filtered signal is closest to the time-frequency spectrum of the original signal shown in Figure 4a.

### 3.2. Structural Damped Free Vibration Signal

**Figure 5:**
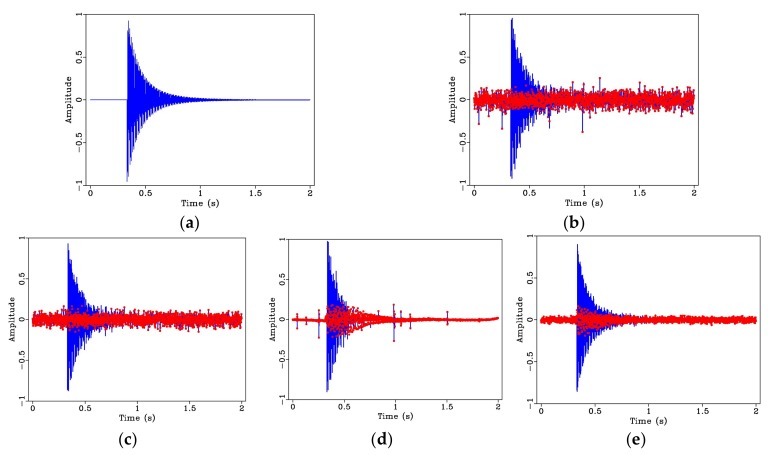
Synthetic structural damped free vibration signal and denoising effects: (**a**) Original signal; (**b**) original noisy signal; (**c**) signal using median filtering; (**d**) signal using wavelet denoising, and (**e**) signal using bilateral filtering. The dotted red line represents the residual noise.

Compared with the dual-frequency chirp signal, a structural damped free vibration signal is composed of a series of sine functions. Each harmonic frequency has a different amplitude value; the composite signal can better simulate the acceleration response signal generated by vibration in structural damage detection. Figure 5a shows the synthetic damped free vibration signal having a fundamental frequency, *f_n_* = 115 Hz and five sub-harmonic frequencies, with a signal duration of 2 s. In Figure 5b, which shows the noisy version of the signal, noise interference with large amplitude peaks appear in the signal, which seriously affects the data quality for subsequent processing. As seen in Figure 5c, after denoising using median filtering, the noise interference with large amplitude values can be effectively suppressed, but this technique is not effective for removing random noise with zero-mean value. The result of wavelet denoising is displayed in Figure 5d. Here, on the contrary, wavelet denoising is able to remove zero-mean random noise, but it is not effective for random peaks. The resulting signal when bilateral filtering is employed is shown in Figure 5e. Although there is still a small amount of background noise in the signal, the overall shape of the signal is closer to the original signal without noise. The bilateral filter parameters used are: R = 15, $\sigma_{d}$ = 10, and $\sigma_{r}$ = 0.1.

Next, the SNR of the original noisy signal and the denoised signal by three denoising techniques are calculated and are displayed in Table 2. The SNR of the signal is obviously improved after processing by median filtering and wavelet denoising method. Nevertheless, the proposed bilateral filtering technique suppresses more noise interference, achieving the highest SNR.

**Figure 6:**
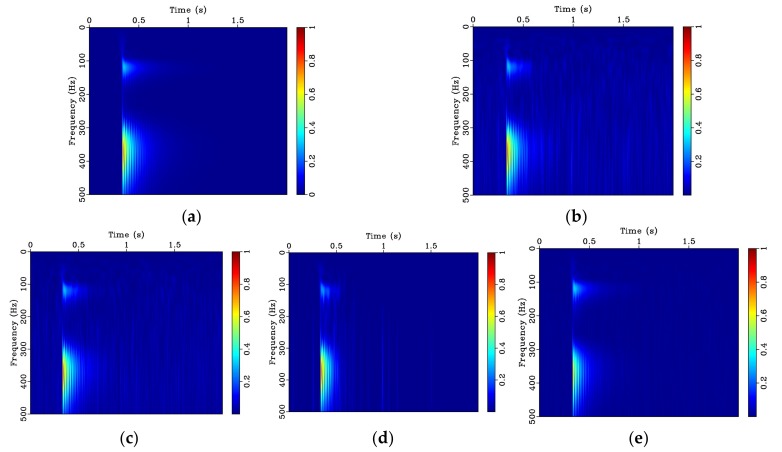
Time-frequency spectrum for simulated damped vibration signal with random noise and denoising results: (**a**) spectrum for original signal; (**b**) spectrum for original noisy signal; (**c**) median filtering result; (**d**) wavelet denoising result and (**e**) bilateral filtering result.

Similarly, time-frequency analysis based on the *S* transform is performed for comparison. After the random noise is added, the noise is significantly disturbed in the 0–500 Hz range of the time-frequency spectrum as shown in Figure 6b. The result of median filtering denoising is displayed in Figure 6c. Noise interference in the time-frequency spectrum is reduced; however, the amplitude value of the noise is still high throughout the frequency band. Wavelet denoising further improves the result, as is shown in Figure 6d, but there are still some random peak bands present. The result after bilateral filtering is employed shows the best improvement in the overall quality of the signal, as can be observed in Figure 6e.

## 4. Evaluation Using Experimental Data

In this section, data recorded from a non-destructive test of a laboratory-scale reinforced concrete beam are processed using different filtering techniques and compared.

### 4.1. Test Setup and Procedure

**Figure 7:**
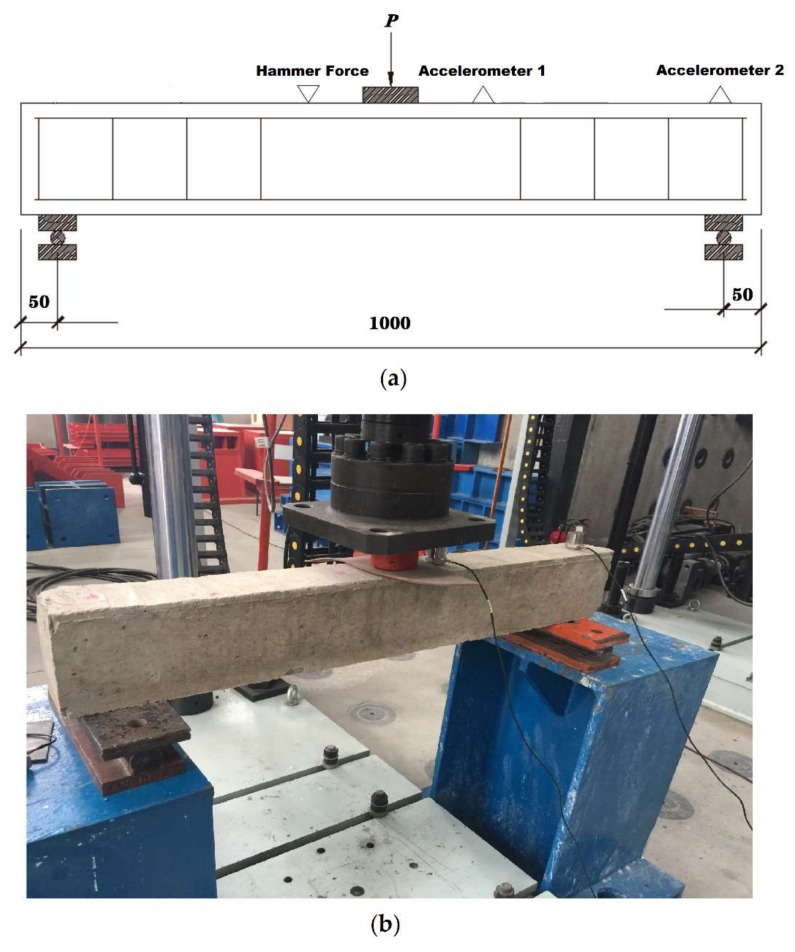
(**a**) Illustration of test setup with reinforced concrete beam and (**b**) photograph showing experimental setup. Dimensions in (mm).

In a non-destructive test, a simply supported reinforced concrete beam was excited by striking an instrumented hammer with a rubber tip at a designated impact location on the beam. A hydraulic actuator with a load cell was used to apply several different vertical loads, *P* at mid-span to induce different stages of cracking. Figure 7 shows the test setup and instrumentation. The overall length of the beam is 1000 mm with a span length of 900 mm. The cross-sectional dimensions are b × h = 100 mm × 150 mm. The longitudinal bars in the compression zone are 2 Ø6 mm, the longitudinal bars in the tension zone are 2 Ø10 mm, and the stirrups are Ø6 mm spaced @ 100 mm. The concrete used for the beam was C20. The impact hammer is made by Jiangsu Donghua, Model DH118. The vibration response was recorded by two accelerometers located on top of the beam: Accelerometer 1 was located 50 mm to the right of mid-span and Accelerometer 2 over the right support. The piezoelectric accelerometers are manufactured by Jiangsu Donghua, Model DH105E and have a sensitivity of 100 mV/ms^2^ and frequency response range of 0.1 Hz to 1 kHz. 

**Figure 8:**
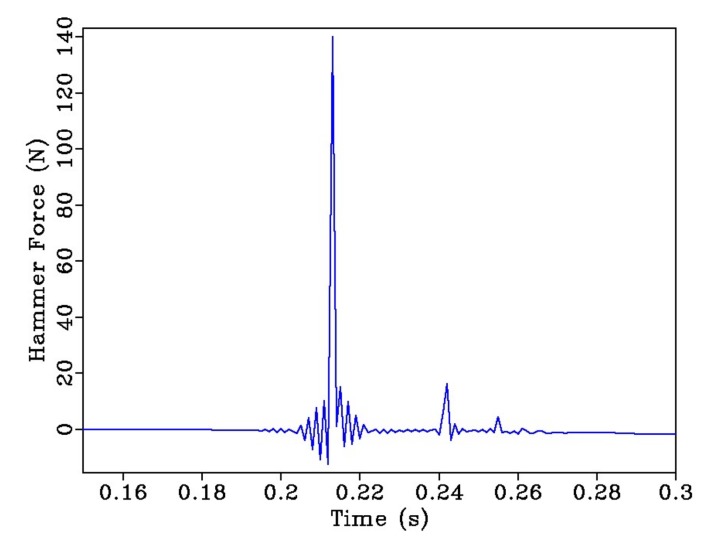
Sample of a typical hammer impact signal.

The hammer impact location is located 50 mm away to the left of mid-span. In order to ensure consistency of the impact source, the force amplitude was kept at 140 ± 10 N. A typical hammer impact signal is displayed in Figure 8.

### 4.2. Signal Processing and Results

The experiment was carried out in a relatively open environment. During the experiment, the measurements were subjected to environmental noise, internal electrodes of the instrument, and noise generated by the power amplifier, thus reducing the quality of the recorded data. Increasing loads, *P* applied at mid-span were used to introduce higher levels of cracking. After each load was applied, it was held for 2 min followed by unloading. After each unloading phase, hammer impacts were performed on the beam and the vibration response recorded by the two accelerometers. In this article, impact tests associated with the following applied load stages are discussed further: 5, 15, 25, and 35 kN.

**Figure 9:**
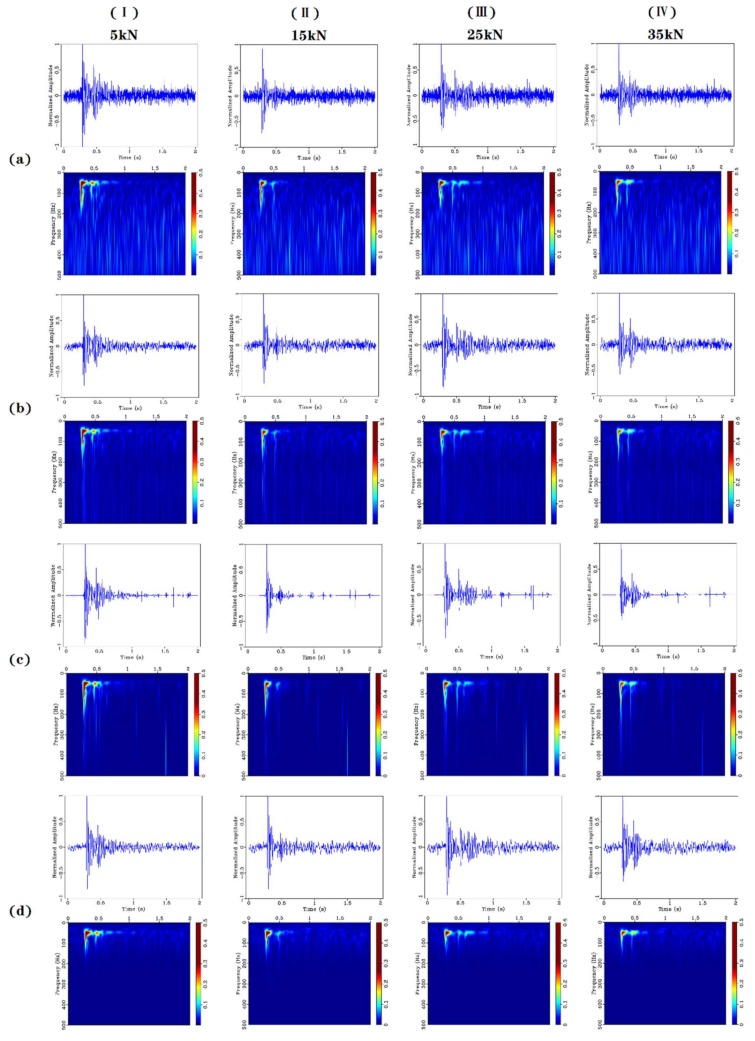
Time domain signals and corresponding time-frequency domain results for each loading stage for Accelerometer 1: Columns (**I** to **IV**) show the loading stage and the rows correspond to the filtering technique: (**a**) Spectrum for original signal; (**b**) median filtering result; (**c**) wavelet denoising result and (**d**) bilateral filtering result.

In Figure 9, the results for Accelerometer 1 are shown. Rows (a) to (d) show both the vibration response in the time-domain as well as the corresponding time-frequency spectrum. Columns (I) to (IV) correspond to load stages 5 to 35 kN, respectively. It can be seen from the time-frequency spectrum in that with increasing load stage, the amplitude of the signal gently decreases. This can be explained by the appearance and extension of cracks inside the beam with increasing load. Cracking causes the energy of the elastic wave to decrease as it interacts with cracks. However, it is difficult to extract useful and quantitative information from the time-frequency spectrum due to the presence of random noise.

**Figure A1:**
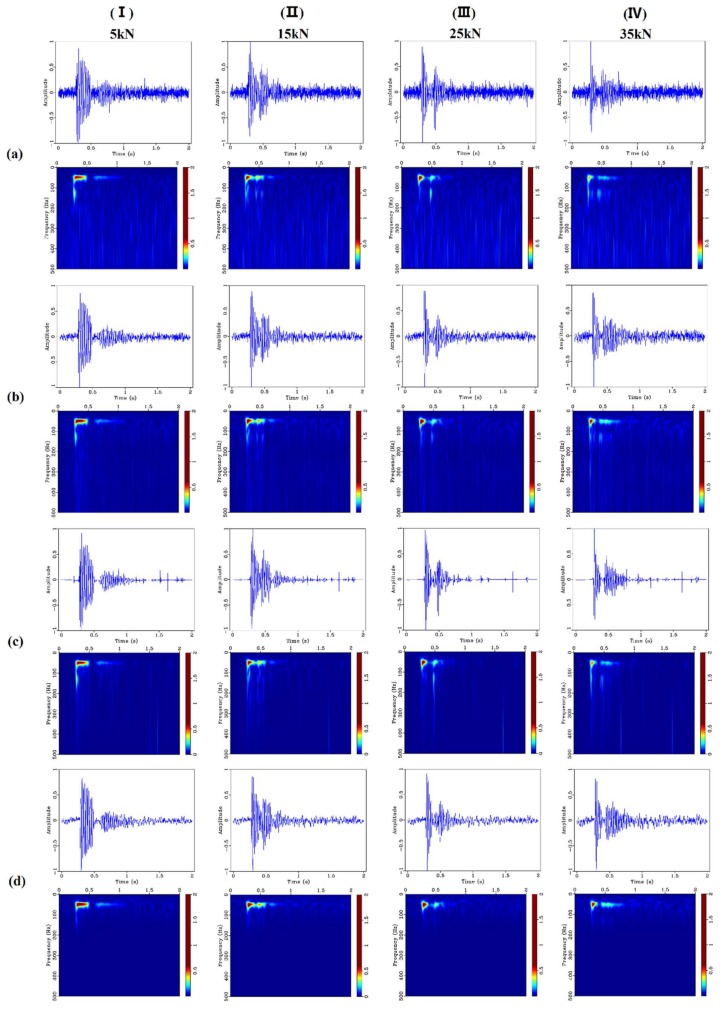
Time domain signals and corresponding Time-frequency domain results for each loading stage.

The signals collected from Accelerometer 2 are not discussed in this paper; the processed results are shown in Figure A1 in the Appendix A.

Based on the above analysis of the measured vibration response signals, median filtering, wavelet denoising and bilateral filtering were used to suppress the random noise. The denoising result of the median filtering as shown in Figure 9b, the noise interference with larger amplitude is suppressed, but the random noise interference is not effectively processed. It can be seen from the time-frequency spectrum that the noise amplitude value is still distributed over the entire frequency band and it is hard to recognize whether the simply supported beam is damaged or not from the time-frequency spectrum. Figure 9(cI) to (cIV) show the results for wavelet denoising processing. Evidently, the wavelet denoising method can suppress the random noise interference better, but the noise with larger amplitude cannot be effectively removed. These large amplitude noise interferences do not only appear in time domain signals, they are easy to observe in the time-frequency spectrum as well. It is, however, difficult to distinguish between effective signal and noise interference. Especially for loading stage IV (35 kN), the high-frequency signal should be rapidly attenuated when the elastic wave passes through the cracked concrete beam, but the signal still has a frequency component above 200 Hz (see Figure 9(cIV)). Compared with the median filtering and wavelet denoising methods, the bilateral filtering method has a better denoising effect on different types of noise interference, the denoising results presented in Figure 9d. It can be seen from the time-frequency spectra that not merely the noise amplitude of the high frequency part are removed, but also the energy concentration area of the time-frequency spectra show a reasonable attenuation trend with the gradual increase of the loading force, which means as the loading force increases, cracks steadily appear in the simply supported beam. At the same excitation level, the energy attenuation increases as the elastic wave propagates; accordingly, in the time-frequency spectra, the energy concentration area decreases gradually.

**Figure 10:**
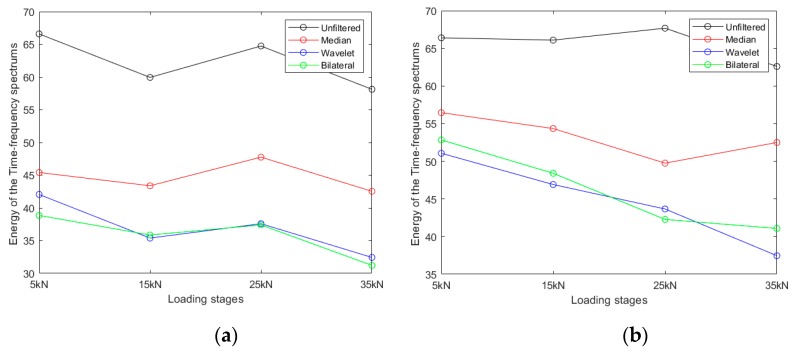
Time-frequency energy *E* for the different denoising approaches: (**a**) results for Accelerometer 1; (**b**) results for Accelerometer 2.

Time-frequency energy *E* was calculated next using Equation (21) for each recorded signal and plotted against load stage (5 to 35 kN). For Accelerometer 1 (Figure 10a), it can be observed that overall, independent of the filter was used, *E* decreases with increasing load stage. From 15 and 25 kN, however, *E* actually increases, which makes *E* not a viable parameter usable for detecting cracking. Since the original signal contains a large amount of noise interference, the calculated energy value *E* is much higher. The calculated value for the median filtering method and the wavelet denoising method are much smaller, and the overall *E* value calculated by the wavelet denoising method is relatively lower. Because the bilateral filtering method most effectively suppresses excessive random noise interference, *E* has the lowest values. For Accelerometer 2 (Figure 10b), the overall trend of the data is more consistent compared to Figure 10a. For this case, both wavelet and bilateral filtering produce curves with a consistent negative slope, which, in turn, are consistent with higher levels of cracking.

**Figure 11:**
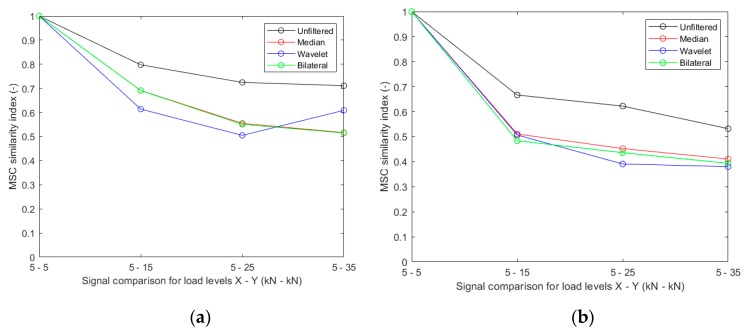
Magnitude-squared coherence (MSC) values for different denoising approaches: (**a**) results for Accelerometer 1; (**b**) results for Accelerometer 2.

Figure 11 shows the results when magnitude-squared coherence (MSC) is used to compare the time response histories. MSC operates in the frequency domain, producing a function of normalized coherence vs. frequency. In this case, the integral of the MSC function was used, which produces a value ranging from 0 to 1, as a measure of similarity between two signals. For the data studied in this article, the integral was computed from 0 to 0.5*f_Nyquist_*. This idea of using MSC was first proposed by Grosse [29] to compare acoustic emission signals. More recently, the same approach was used by the second author of this article on ultrasonic monitoring data [30]. For Accelerometer 1, it can be observed that only wavelet and bilateral filtering produce a consistent trend for the MSC similarity index with increasing cracking. The results for Accelerometer 2 are consistent for any filtering method but most clear for the data denoised by bilateral filtering.

## 5. Summary and Conclusions

In this article, a new signal denoising approach based on bilateral filtering is presented and evaluated on both synthetic and experimental signals. Bilateral filtering is typically used in image processing, having excellent edge-preserving properties. More traditional filtering schemes including median filtering and Wavelet filtering were performed for comparison. The *S* transform was used as an auxiliary method to effectively compare and verify the denoising effect of the different filtering approaches visually. The performance of the three filters are first explored using synthetic signals containing Gaussian noise. A structural vibration test is then presented and discussed. Furthermore, the concept of time-frequency energy is introduced to quantify the change of time-frequency spectra at each loading stage. Finally, the results of magnitude-squared coherence (MSC) for the data from Accelerometer 1 and 2 are shown to exemplify the effectiveness of the bilateral filtering method for quantifying varying levels of cracking.

Overall, we demonstrated that bilateral filtering offers advantages over traditional schemes, in that it is more effective for noisy signals. Bilateral filtering is particularly effective in removing high-frequency noise in synthetic signals with Gaussian noise. This can be observed in the highest SNR and lowest energy in the filtered signals. For the laboratory vibration test, both median and bilateral filtering allowed for producing a consistent indicator of cracking in the beam. The bilateral filtering method was most effective in removing noise interference and random noise interference with large amplitude. 

Future work includes analyzing impulse response data from a large-scale laboratory concrete test as well as other tests such as acoustic emission or portable ground penetrating radar.

## Figures and Tables

**Table 1:** Signal-to-noise ratio (SNR) of synthetic dual-frequency chirp signals.

Type of Signal	Energy of Signal	Energy of Noise	SNR (dB)
Original noisy signal	1000	99.3	10.0
Denoised signal using median filtering	1000	17.2	17.6
Denoised signal using wavelet denoising	1000	10.9	19.6
Denoised signal using bilateral filtering	1000	9.82	20.1

**Table 2:** SNR of synthetic structural damped free vibration signals.

Type of Signal	Energy of Signal	Energy of Noise	SNR (dB)
Original noisy signal	22.8	6.92	5.18
Denoised signal using median filtering	22.8	4.11	7.45
Denoised signal using wavelet denoising	22.8	2.86	9.02
Denoised signal busing bilateral filtering	22.8	2.71	9.26

## Appendix A

Figure A1 shows the time domain signals and their corresponding time-frequency spectra at each loading stage for Accelerometer 2.
